# Retraction: Promising antimicrobial and antibiofilm activities of reduced graphene oxide-metal oxide (RGO-NiO, RGO-AgO, and RGO-ZnO) nanocomposites

**DOI:** 10.1039/d4ra90147a

**Published:** 2024-12-17

**Authors:** Sherif Elbasuney, Gharieb S. El-Sayyad, Hesham Tantawy, Amr H. Hashem

**Affiliations:** a Head of Nanotechnology Research Center, Military Technical College (MTC), Egyptian Armed Forces Kobry Elkobbah Cairo 262-111 Egypt; b Chemical Engineering Department, Military Technical College (MTC), Egyptian Armed Forces Kobry Elkobbah Cairo 262-111 Egypt; c Drug Radiation Research Department, National Center for Radiation Research and Technology (NCRRT), Egyptian Atomic Energy Authority (EAEA) Nasr City Cairo 11787 Egypt Gharieb.Elsayaad@eaea.org.eg; d Botany and Microbiology Department, Faculty of Science, Al-Azhar University Cairo 11884 Egypt amr.hosny86@azhar.edu.eg

## Abstract

Retraction of ‘Promising antimicrobial and antibiofilm activities of reduced graphene oxide-metal oxide (RGO-NiO, RGO-AgO, and RGO-ZnO) nanocomposites’ by Sherif Elbasuney *et al.*, *RSC Adv.*, 2021, **11**, 25961–25975, https://doi.org/10.1039/D1RA04542C.

The Royal Society of Chemistry hereby wholly retracts this *RSC Advances* article due to concerns with the reliability of the data.

In Fig. 10, panels C and D show the same Petri dishes. The authors state this was due to an error in preparing the manuscript.

In the SEM images of Fig. 13, the insets of Fig. 13a–d do not match the area they are indicating. There is overlap in the SEM images in (b) and (c). In addition, the inset of Fig. 13c was duplicated in a section of another image published by the authors,^1^ but representing a different sample. The replacement images provided by the authors do not support the results that “biofilm growth was limited” or from Fig. 13D that the ‘biofilm mass was controlled”. In addition, the information provided in the collection data at the bottom of each replacement image shows that they have not been collected at a consistent magnification, while the scale bars suggest that they have been, which affects the reliability of the data.

Given the significance of these concerns, the Editor has lost confidence that the findings presented in this paper are reliable.

This retraction supersedes the information provided in the Expression of Concern related to this article.

The authors were informed about the retraction of the article. Amr H. Hashem and Gharieb S. El-Sayyad have not agreed with the decision, the other authors have not responded.

Signed: Laura Fisher, Executive Editor, *RSC Advances*

Date: 2nd December 2024

## References

1 M. I. A. Abdel Maksoud, G. S. El-Sayyad, H. S. El-Bastawisy and R. M. Fathy, *RSC Adv.*, 2021, **11**, 28361–28374.

